# Comments on Ambrogi et al. Lung Metastasectomy: Where Do We Stand? Results from an Italian Multicentric Prospective Database. *J. Clin. Med*. 2024, *13*, 3106

**DOI:** 10.3390/jcm13237142

**Published:** 2024-11-26

**Authors:** Tom Treasure, Fergus Macbeth

**Affiliations:** 1 Clinical Operational Research Unit, Department of Mathematics, University College London, London WC1E 6DE, UK; 2 Centre for Trials Research, Cardiff University, Cardiff CF10 3AT, UK

We were interested to read the results from the Italian database [1]. The authors are appropriately cautious in their conclusions about the effects of lung metastasectomy on survival, reminding readers that it is “a local treatment of a systemic disease”. They also note in their concluding paragraphs that “case selection is based on known favorable prognostic indicators”. This was a core lesson of the PulMiCC study (Pulmonary Metastasectomy in Colorectal Cancer).

We appreciated the citation to the preliminary results of the PulMiCC randomised controlled trial published five years ago [2]. The climate of opinion at the time—that lung metastasectomy was of proven benefit—made randomisation to a non-metastasectomy arm difficult for cancer teams. This entrenched belief was captured in the US Society of Thoracic Surgeons (STS) Expert Consensus Document on Pulmonary Metastasectomy [3]. It included the assumption that survival with lung metastases would be zero without operation. However, by only citing the early report of PulMiCC from five years ago, the authors have made a serious error.

We refer all thirteen authors and their readers to a small but brilliant book *Scienzia, quo vadis?*—the English translation is *The Overproduction of Truth*—by Gianfranco Pachionni, from Università degli Studi di Milano-Bicocca, which addresses several issues around the integrity of scientific publication [4]. In it, the author emphasises the importance of citing relevant prior publications, not only papers that agree with you, but also ones that do not. Since the dominant pathology in the cohort reported by Ambrogi et al. was colorectal cancer, PulMiCC is directly relevant. A quick search would have revealed a number of papers giving more information about the full PulMiCC study.

The results are summarised in the figure published in the *British Medical Journal* in November 2023 [5]. The upper panel shows the survival of 491 patients for whom the cancer teams overrode the randomisation protocol [6]. The survival in this electively operated cohort is very much like that in the Italian database report. However, the survival for patients not having a metastasectomy is a long way from the STS assumption of zero [7].

The 93 patients in the PulMiCC randomised controlled trial are shown in the lower panel. Instead of “case selection … based on known favorable prognostic indicators” [1] the indicators were used to ensure that they were balanced in the randomised arms [8] for both *oncological* indicators—number of lung metastases, carcinoembryonic antigen, time since CRC resection, liver involvement—and *patient* factors—age, sex, ECOG (Eastern Cooperative Oncology Group) scores, %FEV1 (forced expiratory volume in 1st second), and BMI (body mass index). There is no evident effect of operation as the lines overlap each other [9]. The power calculation [10] was based on proving that leaving the metastases is *not inferior* to removing them but if there ever were to be a difference it would have to be small.

The full PulMiCC cohort emphasised the burden of treatments that these patient experience [11] and the RCT showed absence of health utility of metastasectomy [12].

Pacchioni makes another important point in his book. He writes “reproducibility of results has been the foundation of Galilean science—at least until now”. There has been a huge proliferation of observational, non-randomised studies about lung metastasectomy [3]. The International Registry study of Lung Metastases included 5206 patients 27 years ago. [13] With less than 10% of the number of patients, this registry study adds nothing and is an example of “overproduction of truth”. What no one has attempted is to refute PulMiCC by replication rather than by dismissing it in a few lines. Put cautiously, if you believe the difference in survival between metastasectomy and non-metastasectomy is 40% survival versus 5% (35%), the effect size is 0.35. That could be provable with 80% power in an RCT of 44 patients or 90% power with 56 patients [10]. However, to date, colorectal oncology teams have wanted to cling onto “the myth” rather than test it, despite the obvious uncertainty of benefit that is shown by careful reading of all the evidence from PulMiCC.
